# Systemic infection drives urgent care needs and outcome in adults with long-term neurological conditions

**DOI:** 10.1016/j.bbih.2022.100538

**Published:** 2022-10-21

**Authors:** Ana Saldanha Ramos, Ian Galea, Aravinthan Varatharaj

**Affiliations:** a Wessex Neurological Centre, University Hospital Southampton NHS Foundation Trust, UK; b Clinical Neurosciences, Clinical and Experimental Sciences, Faculty of Medicine, University of Southampton, UK

**Keywords:** Long-term neurological conditions, Urgent care, Systemic infections

## Abstract

It is estimated that 1 in 6 people are living with a long-term neurological condition (LTNC). Although it is likely that systemic infections are a common trigger for urgent tertiary care needs in LTNCs, there is a lack of data. Yet this is important since systemic infections are a modifiable risk factor, and hence the motivation for a formal evaluation. We undertook case note review of 155 consecutive unselected adult patients with LTNC receiving urgent care at a tertiary hospital between November and December 2019. Data were collected on presenting symptoms, diagnosis, length of stay, complications, and change in social needs. The most common LTNCs were neurocognitive disorders (n = 68, 44%), cerebrovascular disorders (n = 65, 42%), and epilepsy (n = 19, 12%). Respiratory infections were most common (n = 40, 62.5%), followed by urinary (n = 16, 25%), skin (n = 4, 6%), gastrointestinal (n = 3, 5%) and bone (n = 1, 1.5%). Systemic infection was the trigger for urgent care in 41.3% of patients and in multivariable regression was associated with an increased likelihood of admission (p < 10^−5^, OR = 7.8, Nagelkerke R^2^ = 0.37), longer length of stay (p = 0.03, β = 5.91, R^2^ = 0.06), and death (p = 0.045, OR = 4.3, Nagelkerke R^2^ = 0.22). Altered mental status was the presenting symptom most frequently associated with infection (p < 10^−8^, χ^2^ test). In conclusion, systemic infections are a major trigger of acute tertiary care needs in adults with LTNCs, and play a role in determining clinical outcome. Since systemic infections are preventable or can be treated if identified early, they may represent a modifiable target to improve quality of life, clinical outcomes and health service efficiency.

## Highlights

• Long-term neurological conditions (LTNC) have a high mortality, morbidity and cost.
• We studied the role of systemic infection in LNTC admissions for tertiary care.
• Systemic infection was the trigger for urgent care in 41.3% of LTNC patients.
• It was associated with admission to hospital, longer length of stay and death.
• Systemic infection is a modifiable target to improve outcomes in LTNC.

## 1 Introduction

It is estimated that 1 in 6 people in the UK are living with a long-term neurological condition (LTNC) (The Neurological Alliance, 2019), defined as “a disease of, injury or damage to the body's nervous system which will affect the individual and their family in one way or another for the rest of their life” (Department of Health and Social Care, 1981). LTNCs are a leading cause of disability and death (Deuschl et al., 2020), and contribute significantly to healthcare expenditure (Olesen et al., 2012).

There are little data on the drivers for urgent care in people with LTNCs. In particular, systemic infection (outside the central nervous system) is widely recognised as a common cause of acute deterioration, but its impact on urgent care utilisation and outcomes is not well studied. This is particularly important since many infections are preventable or can be treated if identified early. Moreover, people with LTNCs may be more vulnerable to developing systemic infections (for example, aspiration pneumonia in stroke), as well as being more prone to its neurological effects such as delirium (Ahmed et al., 2014). Thirdly, systemic infections may cause short-term deterioration of LTNC symptoms, and potentially drive long-term disease progression in some LTNCs such as Alzheimer's disease (Holmes et al., 2009). Finally, infections may be more difficult to diagnose in people with LTNCs, due to non-specific presentation, absence of fever, communication difficulties, and confounding effects of drug treatment (Ahmed et al., 2014).

## 2 Methods

A single assessor (ASR) reviewed all adults (age above 18) referred for urgent care at University Hospital Southampton NHS Foundation Trust, a large teaching hospital providing care to 1.9 million people. The inclusion criterion was an existing LTNC as per the National Service Framework definition (Department of Health and Social Care, 1981). Conditions were identified as being neurological if they were included in The Neurological Alliance's 2019 report on neurological conditions (The Neurological Alliance, 2019). In conditions not expressly included in this report, inclusion was determined by consensus amongst senior authors (AV and IG). Diagnoses were clustered according to the relevant ICD-11 two-character codes (World Health Organization, 2022). Data were collected on 18 randomly selected days, including weekends, over the course of November and December 2019, using a standardised proforma. Urgent care assessments, previous discharge summaries, drug history, correspondence, and online primary care records were reviewed. The possible outcomes were discharge, admission to an inpatient ward, or death. Data were gathered on presenting symptoms, acute diagnosis, neurology specialty input, length of stay, mortality, and change in care needs (defined as the requirement for a new or increased package of care, or transfer to a rehabilitation hospital). Institutional approval was obtained on the basis of a service evaluation (SEV/0443).

Analyses were performed in SPSS version 26 (IBM Corporation). Histograms and Kolmogorov–Smirnov tests were used to assess normality. Comparison between groups was by two-tailed parametric or non-parametric tests as appropriate. Linear or logistic regression was used to assess variables as predictors of admission, length of stay, increased care needs, and mortality. A p-value < 0.05 rejected the null hypothesis.

## 3 Results

659 individuals received urgent care during the study, of whom 155 (23.5%) had a LTNC and were eligible for inclusion. All LTNC diagnoses were those recognised by the Neurological Alliance, except for one individual with neurosarcoidosis, included by consensus. One individual with uncomplicated Bell's palsy fully recovered five years prior was excluded. There were no missing data. Two individuals died whilst being assessed prior to admission, and were excluded from analyses of admission likelihood. In total 11 individuals died, and all were excluded from analyses of increasing care needs.

**Fig. 1:**
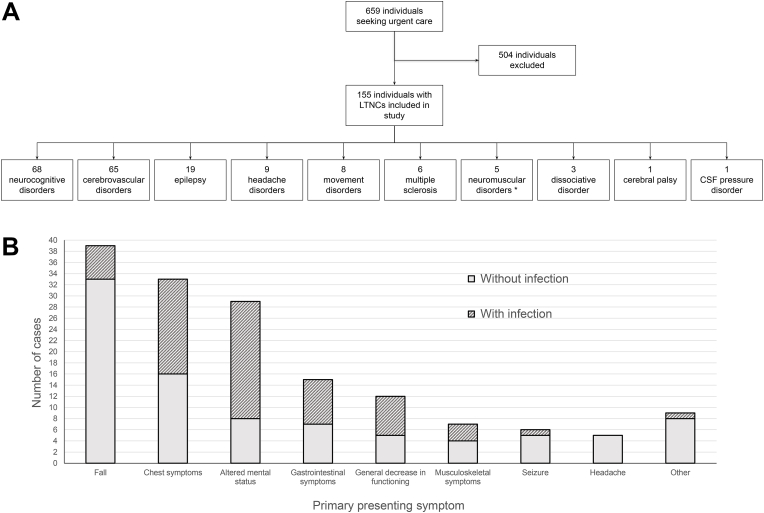
**A:** Breakdown of eligible individuals by LTNC two-character ICD-11 category. Since many individuals had multiple diagnoses, the sum of numbers in the bottom row is greater than the total number of individuals. * A combination of ICD-11 categories 8B-8D: ‘Motor neuron diseases or related disorders’, ‘Disorders of nerve root, plexus, or peripheral nerves’, and ‘Diseases of neuromuscular junction or muscle’. **B:** Primary presenting symptoms for individuals seeking urgent care.

The breakdown of LTNC by categories is shown in Fig. 1A. The most common categories were neurocognitive disorders, cerebrovascular disorders, and epilepsy. Whilst most individuals had one LTNC in a single category, 28 (18.1%) and 2 (1.3%) had LTNCs in two and three categories respectively. Compared to those with a single category diagnosis, individuals with diagnoses in two or more categories were more likely to be admitted (75.0% vs 45.7%, p = 0.005, χ^2^ test).

Systemic infection was the commonest trigger for urgent care in 64 (41.3%) individuals. The respiratory tract was the most common site of infection (n = 40, 62.5%), followed by urinary (n = 16, 25%), skin (n = 4, 6%), gastrointestinal (n = 3, 5%) and bone (n = 1, 1.5%) sites. In individuals without systemic infection, the trigger for admission varied, with mechanical falls (18.1%) and seizures (4.5%) being the most common.

Presenting symptoms are shown in Fig. 1B. The most common symptoms were falls (25.2%), chest symptoms including breathlessness and chest pain (21.3%), and altered mental status (18.7%). There was a significant relationship between the presenting symptom and the presence or absence of infection (p < 10^−5^, χ^2^ test). The presenting symptom most associated with systemic infection was altered mental status; 72.4% of individuals with this presentation were found to have infection. Compared to those without infection, individuals with infection were more likely to be admitted (74.6% vs 35.6%, p < 10^−5^, χ^2^ test), had a longer length of stay (6 days vs 1 day, p < 10^−6^, Mann-Whitney test), and a higher risk of death (12.5% vs 3.3%, p = 0.03, χ^2^ test). There was no significant difference in the likelihood of increased care needs between individuals with or without infection (45.2% vs 54.8%, p = 0.42, χ^2^ test). Detailed results are presented in Table 1.

**Table 1:** Outcome measures stratified by LTNC two-character ICD-11 category and the presence or absence of infection.

	All LTNCs	Neuro-cognitive	Cerebro-vascular	Epilepsy	Headache disorders	Movement disorders	Multiple sclerosis	Neuro-muscular disorders	Dissociative	Two or more LTNCs
n	155	68	65	19	9	8	6	5	3	28
Age, median (range)	83 (22–100)	88 (69–99)	83 (51–100)	57 (22–91)	61 (23–89)	86 (51–90)	66 (38–79)	75 (25–87)	59 (54–69)	85 (56–91)
Female sex (%)	92 (59.4)	43 (63.2)	30 (46.2)	10 (52.6)	9 (100)	2 (25.0)	3 (50.0)	2 (40.0)	2 (66.7)	9 (32.1)
Systemic infection (%)	64 (41.3)	29 (42.6)	29 (44.6)	4 (21.1)	1 (11.1)	4 (50.0)	5 (83.3)	4 (80.0)	1 (33.3)	12 (42.9)
Admission required (%)										
*In all cases*	79 (51.0)	38 (55.9)	42 (64.6)	8 (42.1)	3 (33.3)	3 (37.5)	3 (50.0)	1 (20.0)	3 (100)	21 (75.0)
*In cases with infection*	47 (73.4)	25 (86.2)	21 (72.4)	3 (75.0)	1 (100)	3 (75.0)	3 (60.0)	1 (25.0)	1 (100)	10 (83.3)
*In cases without infection*	32 (35.2)	13 (33.3)	21 (58.3)	5 (33.3)	2 (25.0)	0	0	0	2 (100)	11 (68.8)
Length of stay, median (range)										
*In all cases*	2 (1–174)	4 (1–31)	4 (1–56)	2 (1–23)	1 (1–7)	2 (1–12)	2 (1–174)	2 (1–3)	5 (3–8)	6 (1–31)
*In cases with infection*	6 (1–174)	1 (1–30)	6 (1–56)	3 (2–23)	7 (7–7)	9 (2–12)	3 (1–174)	3 (2–3)	3 (3–3)	6 (1–31)
*In cases without infection*	1 (1–44)	8 (1–31)	4 (1–44)	2 (1–19)	1 (1–7)	1 (1–2)	1 (1–1)	1 (1–1)	7 (5–8)	6 (1–19)
Increase in care needs (%)										
*In all cases*	31 (21.5)	16 (25.3)	16 (28.1)	1 (5.3)	1 (11.1)	2 (28.6)	1 (16.7)	1 (20.0)	0	7 (28.0)
*In cases with infection*	14 (25.0)	7 (29.2)	4 (17.4)	1 (25.0)	0	2 (50.0)	1 (20.0)	1 (25.0)	0	2 (22.2)
*In cases without infection*	17 (19.3)	9 (23.1)	12 (35.3)	0	1 (12.5)	0	0	0	0	5 (31.3)
Died (%)										
*In all cases*	11 (7.1)	5 (7.4)	8 (12.3)	0	0	1 (12.5)	0	0	0	3 (10.7)
*In cases with infection*	8 (12.5)	5 (17.2)	6 (20.7)	0	0	0	0	0	0	3 (25.0)
*In cases without infection*	3 (3.3)	0	2 (5.6)	0	0	1 (25.0)	0	0	0	0

Cerebral palsy and CSF pressure disorders are not included in this table due to the presence of only one case each. Neuromuscular disorders included conditions affecting the neuromuscular junction such as myasthenia gravis. Individuals who died are excluded from the tabulation of increased care needs.

Multivariable regression was performed to identify characteristics associated with admission, increasing care needs, death, and length of stay. Age, sex, LTNC category (coded as single categories or ‘multiple’ for those with two or more), and the presence or absence of systemic infection were included as independent variables. In binary logistic regression entering all variables, infection significantly predicted admission (p < 10^−5^, OR = 7.8, 95% CI 3.29–18.67, Nagelkerke R^2^ = 0.37) and death (p = 0.045, OR = 4.3, 95% CI 1.03–18.00, Nagelkerke R^2^ = 0.22). No other independent variables reached significance. The likelihood of increased care needs in survivors was not predicted by infection (p = 0.82, OR = 1.1, 95% CI 0.45–2.71, Nagelkerke R^2^ = 0.15). In a linear regression with the same variables, infection significantly predicted increased length of stay (β = 5.91, p = 0.03, R^2^ = 0.06).

## 4 Discussion

Patients with LTNCs made up nearly one-quarter of those seeking urgent care in this study. Systemic infection, primarily affecting respiratory and urinary sites, was the most common trigger for urgent care. Individuals with infection were more likely to be admitted, stay in hospital longer, and die. These observations underscore the importance of systemic infection as a driver for urgent care needs for people with LTNCs. Previous studies have looked at the causes for admissions in geriatric populations (Eriksen et al., 2022; Lin et al., 2016); this study is novel since it investigates people with LTNCs and links systemic infections with outcome.

Altered mental status was strongly associated with systemic infection in this cohort, highlighting this presentation as one warranting a high index of suspicion for occult infection. Systemic infection is a well-recognised cause of delirium, which arises due to an interaction between brain vulnerability and an acute stressor, and so is especially relevant for individuals with LTNCs (Wilson et al., 2020). Relevant mechanisms during infection include systemic inflammation, hypoxia, impaired tissue perfusion, and metabolic derangement, which lead to disturbances of neurotransmission, microglial and astrocytic activation, and blood-brain barrier dysfunction. Identification of delirium is essential for effective management of the acute illness, and also has consequences for long-term cognitive decline (Goldberg et al., 2020). Future work should examine whether the presence of delirium (or sub-syndromic altered mental status) contributes to the early diagnosis of systemic infection independent of other symptoms and signs, as well as exploring novel methods for early detection of delirium such as assessment by family or caregivers (Steis et al., 2012).

The importance of systemic infection as a driver for urgent care needs in adults with LTNCs has been highlighted by COVID-19, and this study shows that the problem predates the pandemic and can be expected to continue without an integrated strategy for the prevention, diagnosis, and treatment of infection. In advanced chronic neuro-degenerative diseases, systemic infections are almost an inevitable consequence of the underlying condition. Numerous practical challenges exist and require solutions. Prevention will include breaking barriers to vaccination uptake and addressing modifiable risk factors for infection in people with LTNCs. For example, impairments in swallowing and bladder function are common across LTNCs and may predispose to respiratory and urinary tract infections respectively. Nutritional status, mobility, and ability to self-care may also represent modifiable targets for prevention. Early diagnosis and treatment of infection is relevant in the community and across urgent care and must be considered in balance with the principles of antibiotic stewardship; this is challenging in practice and advances in this area are needed. Biomarkers and point-of-care tests to rapidly differentiate bacterial from viral infections may be helpful, such as combined C-reactive protein and myxovirus resistance protein A immunoassay (Self et al., 2017), or human neutrophil lipocalin assay (Venge et al., 2019). Long-term non-invasive monitoring of systemic inflammation in people with LTNCs may be possible with regular urinary neopterin measurements (Stuart et al., 2019). A bidirectional training need exists, in that professionals caring for people with LTNCs need to be equipped with the skills to identify and manage systemic infections, whilst professionals in urgent care need the skills to care for people with LTNCs, who represent a significant proportion of admissions. Importantly, people with LTNCs should be supported to best advocate for their own care, including by education to recognise early symptoms of infection. It is important to note that data were collected over winter when respiratory infections are more prevalent – this is when tertiary hospitals come under most pressure and therefore interventions to prevent infections or treat them early would have most benefit in this period.

This retrospective study has several limitations. Our classification of systemic infections rested on diagnoses made by experienced urgent care physicians. However, we did not examine the evidence leading to these diagnoses, and it is possible that both over- and under-diagnosis occurred. For example, there is evidence that clinicians tend to over-diagnose UTI in older adults (Kistler et al., 2022). To address these limitations, a future prospective study should include *a priori* case definitions for systemic infections, comprising clinical and para-clinical markers. Also, presentations such as altered mental status are often multifactorial in origin, especially in older adults. Systemic infections might be just one step in a sequence of events wherein people with LNTC are prone to develop complications which beget more complications such as systemic infections, which ultimately affects outcome in a complex way. As this was a retrospective study focused on systemic infections, we did not obtain this more detailed information. We did not examine non-infective stimuli, many of which may lead to a systemic inflammatory response and therefore similar biological effects. For example, hip fracture is commonly associated with delirium, and the role of systemic inflammation here is a topic of ongoing study (Leavey et al., 2019). Hence prevention of injuries or fractures may be as important as the prevention and management of systemic infections. Finally, it is important to note that the findings of this study may not be specific for LTNCs, and may apply to other patient populations.

In conclusion, this study demonstrates that systemic infection is an important driver of urgent care needs for adults with LTNCs. This finding is important since systemic infections are modifiable with potential to improve quality of life and clinical outcomes for people with LTNCs, and make cost savings in health services.

## Author contributions

AV and IG conceived the study. ASR collected the primary data. All authors contributed to analyses, interpretation and manuscript writing.

## Declaration of competing interest

The authors declare the following financial interests/personal relationships which may be considered as potential competing interests: Aravinthan Varatharaj reports financial support was provided by 10.13039/501100000265Medical Research Council.

## Data Availability

Data is available subject to ethical and/or institutional approvals.
